# Waiting is worthwhile: ROP stage 5 with stage regression due to retinal reattachment after scleral buckling surgery (encircling band) – a case report with review of the literature

**DOI:** 10.1186/s12886-025-04397-x

**Published:** 2025-09-23

**Authors:** A. Khamees, V. Schöneberger, S. Kaya, T. Guthoff, G. Geerling, R. Guthoff

**Affiliations:** 1 Department of Ophthalmology, Heinrich-Heine University Düsseldorf, Universitätsaugenklinik Düsseldorf, Düsseldorf, Germany; 2 Faculty of Medicine, Mutah University, Karak, Jordan

**Keywords:** Retinopathy of prematurity (ROP), Scleral buckle surgery, Retinal detachment, Persistent subretinal fluid

## Abstract

**Background:**

Retinopathy of Prematurity (ROP) can progress despite standard treatments such as laser coagulation and VEGF inhibitors. Scleral buckle surgery (SBS) is a surgical option in stage 4 ROP, though anatomical success in stage 5 is rare. We present a unique case of spontaneous retinal reattachment after SBS in stage 5 ROP without further intervention.

**Case presentation:**

A female infant, born at 24 + 2 weeks and weighing 440 g, developed stage III ROP with plus disease in the left eye. Despite laser treatment, the disease progressed to stage 4B, prompting SBS at 39 weeks PMA. Within three weeks, the eye advanced to stage 5 ROP. Surprisingly, complete retinal re-attachment occurred spontaneously within four months. Although the anatomical outcome was favorable, the left eye developed high myopia and amblyopia.

**Conclusions:**

This case highlights the potential for spontaneous improvement after SBS in advanced ROP. While anatomical success is possible, functional outcomes may remain poor. Individualized follow-up and further research on subretinal fluid dynamics are needed to optimize treatment strategies.

## Background

Retinopathy of Prematurity (ROP) is a leading cause of childhood blindness worldwide. The possible therapy options of VEGF inhibitors and/or laser coagulation cannot always prevent progression beyond stage 3. Scleral Buckle Surgery (SBS) has emerged as an alternative treatment modality for advanced stage ROP, offering potential benefits in terms of improving anatomical outcomes and preserving visual function. We report on a 440-mg infant born at 24 weeks and 2 days postmenstrual age (PMA), who underwent complete retinal re-attachment for stage V ROP within four months following laser coagulation and encircling band surgery.

### Case presentation

We present a case study of a 440 g female infant, the second child of a 39-year-old woman, who was born with multiple comorbidities primary related to prematurity (Respiratory Distress Syndrome grade III, apnoea-bradycardia syndrome, metabolic acidosis, hyperbilirubinemia, temperature dysregulation, being small for the gestational age, hyponatremia, hyperglycemia, persistent ductus arteriosus, anemia), primary diseases (intrauterine CMV infection, hypothyreosis, sepsis, ovarian cysts, nephrocalcinosis) and secondary complications (enterobacter infection). The infant was delivered by emergency caesarean section at 24 weeks and 2 days gestational age due to maternal preeclampsia and HELLP syndrome.

The mother received RDS (neonatal respiratory distress syndrome) prophylaxis twice before the delivery. The infant, who was admitted to the NICU for 3 months, received CPAP ventilation and surfactant treatment with less invasive surfactant application among other neonatal care measurements.

**Fig. 1:**
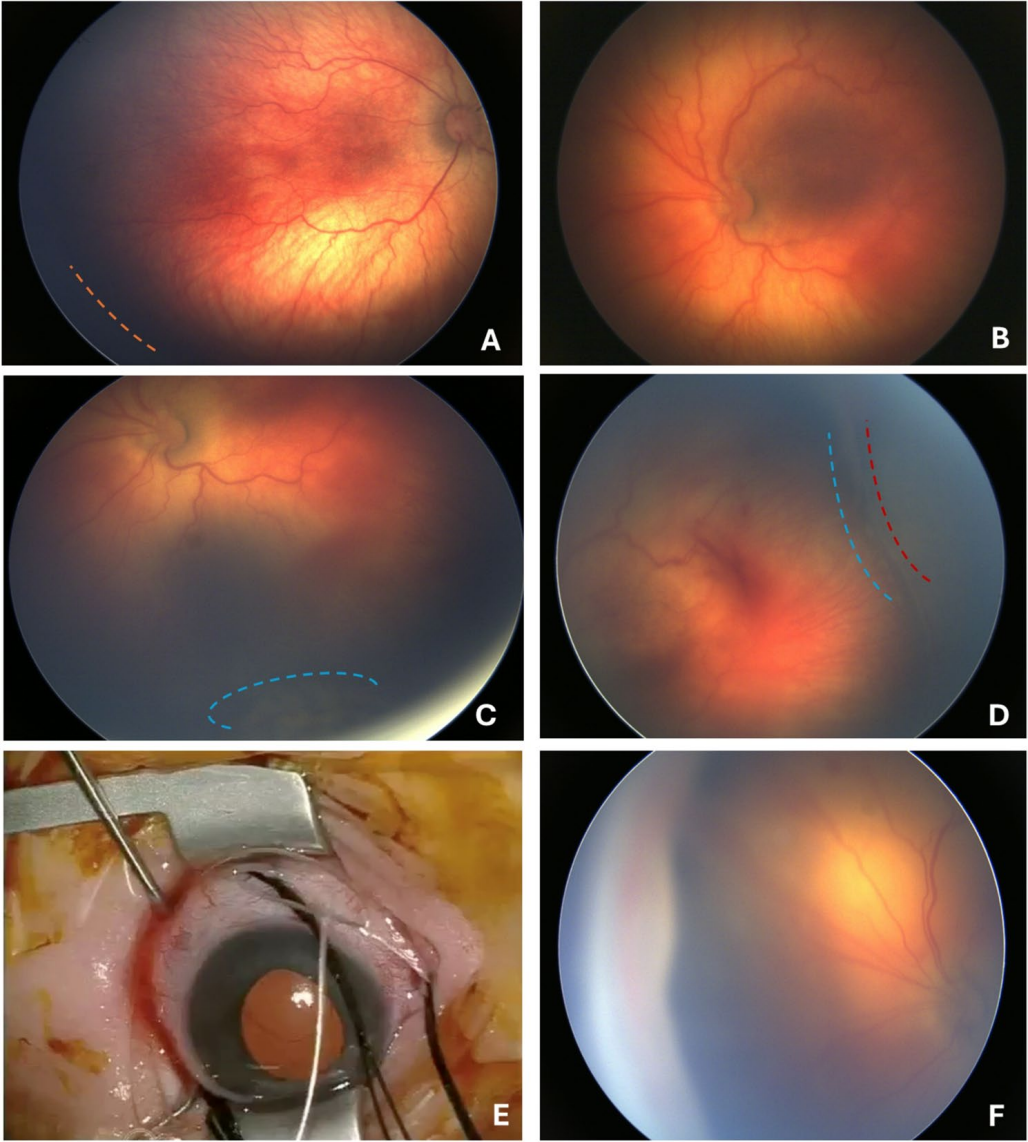
Central wide field fundus image at 36 + 4 weeks of postmenstrual age with zone 2, stage 3 ROP right eye (**A**) and left eye (**B**), visible ridge marked with orange dot line. Two days after laser coagulation with visible foci below with slight retinal detachment marked with blue dot line (**C**), new vitreous haemorrhages and flat but increasing retinal detachment, marked with red dot line (**D**), which is why a 2.5 mm encircling band (Type 240) was sutured on at 39 weeks of PMA (**E** +** F**). Fundus photography was performed using the RetCam (130° field of view), Clarity Medical Systems, USA

The patient was transferred to our clinic at a PMA of 36 weeks and 2 days for ROP treatment. Initial dilated fundus examination showed stage 2 ROP (visible ridge) with plus disease (rigid pupil, hazy media, and increased vascular tortuosity) in zone 2 of the right eye (Fig. 1A; all fundus images were aquired by the RetCam; Clarity Medical Systems, USA). The left eye revealed stage III ROP (elevated ridge with proliferation) in zone 2 with Plus disease (narrow pupil, vitreous haemorrhage and increased vascular tortuosity) (Fig. 1B).

Due to the peripheral elevation, we opted for laser photocoagulation instead of anti-VEGF therapy, which could potentially worsen traction. Both options were discussed with the parents, including the advantages and disadvantages as well as the risks, and informed consent was obtained. Laser coagulation of the peripheral avascular retina in both eyes was performed two days after the presentation. Due to the shallow retinal detachment, an effective laser coagulation could only be performed on a limited area of the retina (Fig. 1B). This implied that the patient had actually been in stage 4a before undergoing laser photocoagulation. Despite undergoing laser therapy, the left eye's retinal detachment progressed (Fig. 1C + D). Therefore, an encircling band type 240 was sewn on at 39 PMA (Fig. 1E + F).

**Fig. 2:**
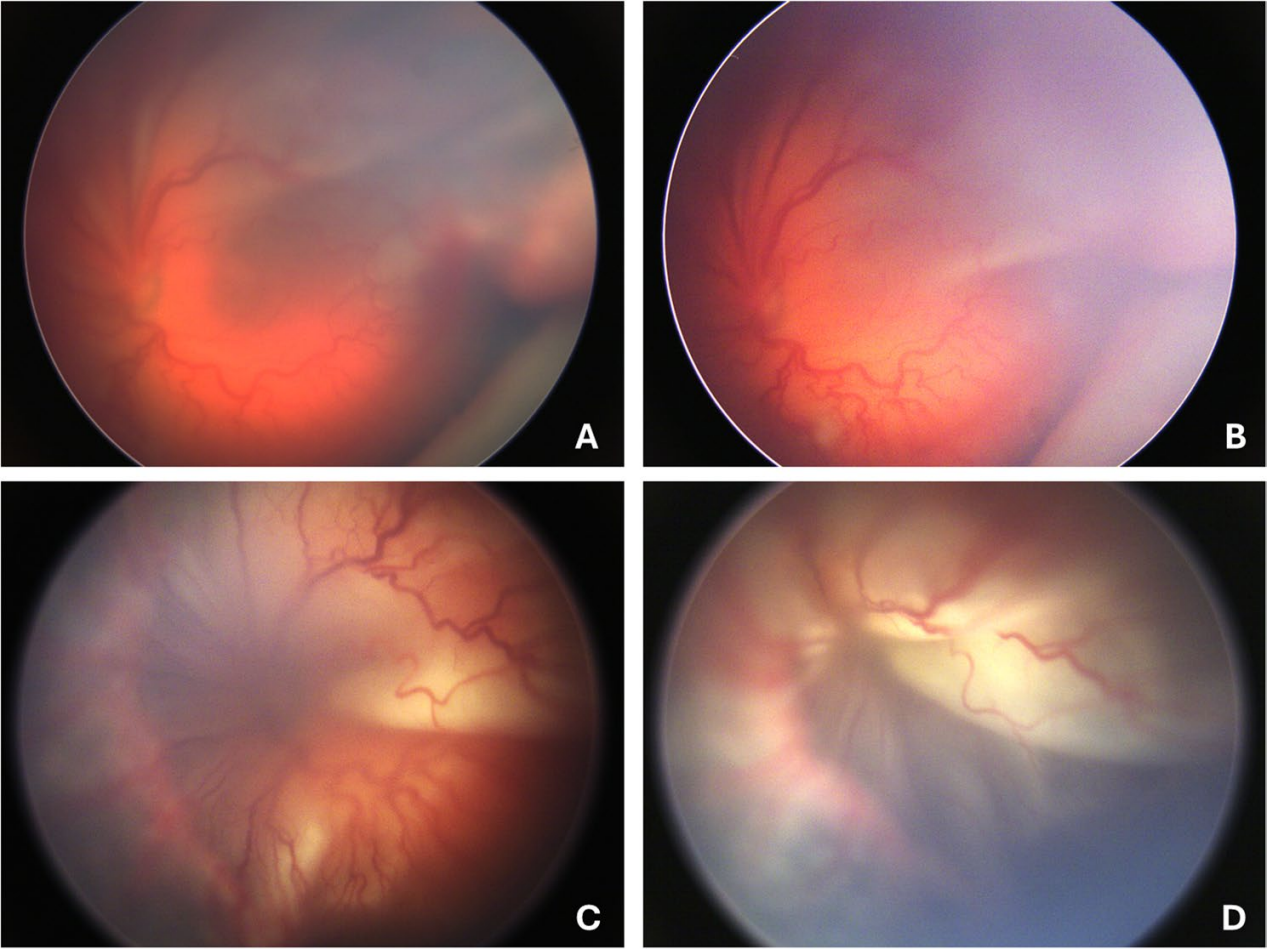
shows retinopathy of prematurity (ROP) progressing to stage 5 within a period of under three weeks after the encircling band was applied. Wide-field image of the left eye, taken five days after the encircling band was applied, shows increased preretinal haemorrhage, as well as superotemporal retinal detachment without macular involvement (**A**). Four days later, the retinal detachment appears stable, but vessel tortuosity and neovascularization increased (**B**). However, almost total retinal detachment with macular involvement is present; ROP stage 4 (**C**). Five days later, ROP stage 5 is shown in (**D**). Fundus photography was performed using the RetCam (130° field of view)

Within one week of scleral buckling in the left eye, there was an increase in preretinal haemorrhage and retinal vessel tortuosity, as well as superotemporal retinal detachment without macular involvement. The retinal detachment progressed to stage 5 within the third postoperative week (see Fig. 2).

**Fig. 3:**
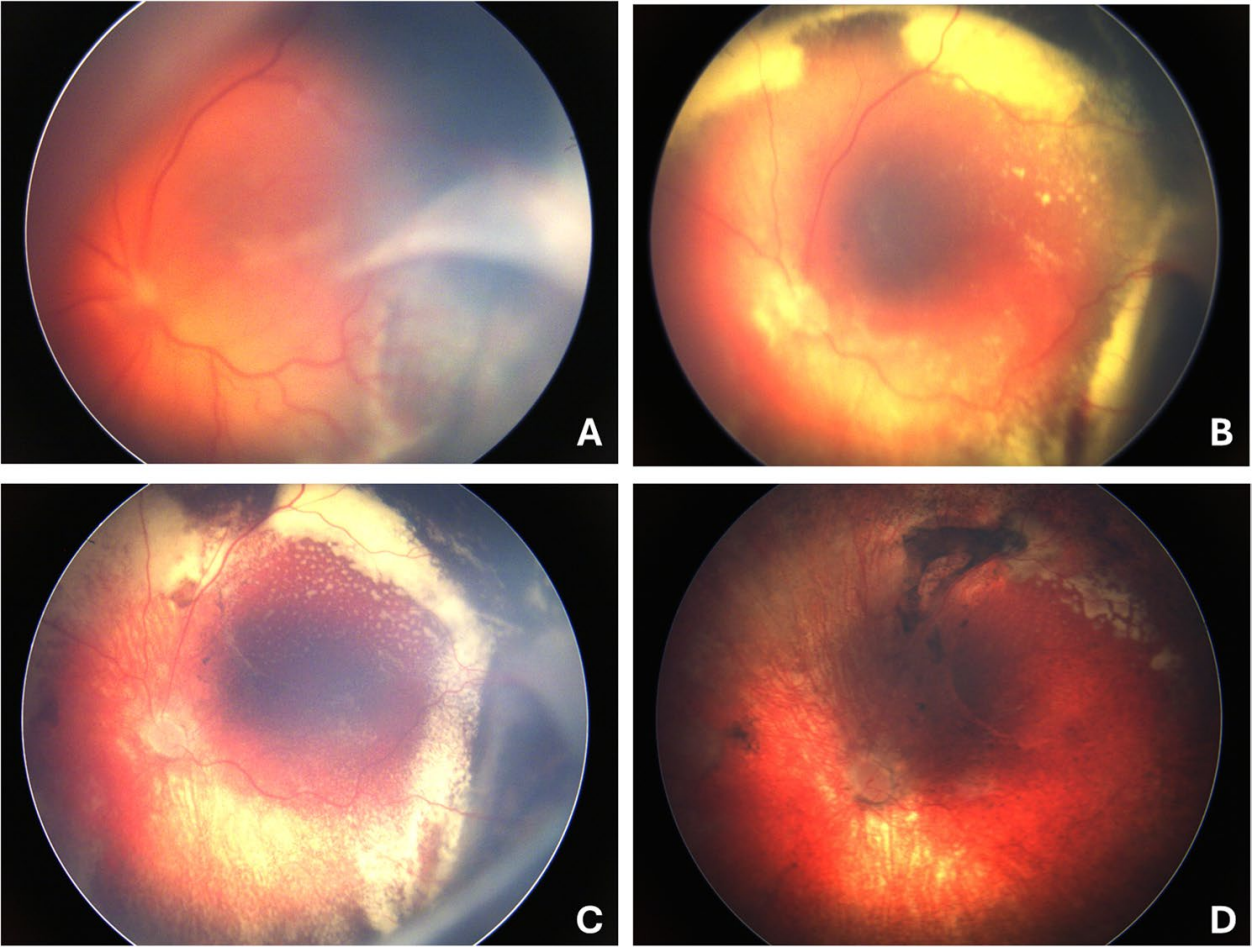
shows wide-field images of the left eye, depicting the chronological changes in retinal detachment and subretinal exudation. Four weeks after the encircling band was applied, a residual retinal fold can be seen in regression (**A**). A further 3.5 weeks later, there is still shallow detachment with resorption and accumulation of hard exudates (**B**). After a further five weeks, spontaneous reattachment of the retinal detachment is seen (**C**). Six months after application of the encircling band, there is also finally a reduction in exudates and scarring (**D**). Fundus photography was performed using the RetCam (130° field of view)

We continued to monitor the patient regularly, and in the eighth week, we observed a significant regression of peripheral subretinal fluid and exudation (Fig. 3). Retinal reattachment was seen at week 16, and the scleral buckle was removed seven months after surgery.

In contrast, the right eye showed age-appropriate development of visual equivalent in preferential looking testing of 0.4 logMAR (Cardiff-Test) by the age of 3 years. Despite wearing glasses with the correct prescription, vision in the left eye was strongly reduced probably due to optic nerve atrophy and amblyopia. Patching of the right eye was not tolerated after the age of 18 months. During the follow-up examination at the age of eight, we performed visual acuity testing using the LEA test for distance vision and the Cardiff test for near vision, due to the patient’s mild intellectual disability. Near vision was better than distance vision in the right eye, with logMAR values of 0.4 and 0.8, respectively with a manifest latent nystagmus. Light perception was observed in the left eye.

## Discussion

Despite of the evidence-based recommendations for screening and intervention, ROP remains a leading cause of preventable childhood blindness and vision loss globally.

Over the past 40 years, significant developments have been made in the treatment for Retinopathy of Prematurity (ROP). Earlier cryotherapy, then laser treatment and most recently, VEGF inhibitor therapy, all of which have improved the chances of preventing disease progression [1]. Nevertheless, according to the pre-VEGF-ETROP Study group, 12% of the treated ROP patients do progress to stages 4 and 5. For these patients, choosing the appropriate treatment is often very challenging. Stage 5 ROP with total retinal detachment is managed surgically, most often with vitrectomy (with or without lensectomy) and occasionally scleral buckling, with reported anatomical success rates of only 20–50%. Even when partial reattachment is achieved, visual outcomes are generally poor [2]. According to one study by Karaçorlu et al., the anatomic success rate after vitrectomy for stage 5 ROP was 42% in 88 eyes, with a mean visual acuity of 20/5000 [3]. Rajan et al. found that the final macular attachment rate in 21 eyes undergoing 25-gauge vitrectomy for stage 5 ROP was 19% [4]. We discussed the option of vitrectomy versus an encircling band in great detail internally before suggesting the latter to the parents. We assumed that vitrectomy, in the case of a very anteriorly displaced retina and the possible necessity of lensectomy, would carry a high risk of phthisis with a subsequent disfiguring facial appearance rather than a chance of preserving vision.

Here, we report a case of rapidly progressive ROP stage 4A for which laser therapy could no Longer be performed adequately. Despite a buckle surgery being performed in stage 4, the condition progressed to stage 5. Surprisingly, it then regressed slowly back to complete retinal attachment without further intervention.

While laser therapy according to the current guidelines remains a valuable tool in managing Zone 2 ROP, it is now part of a broader approach that considers various factors and therapies to provide the best possible care for affected infants. Risk factors for progression from stage 3 zone 2 despite VEGF inhibitors include, but are not limited to, gestational age of less than 29.5 weeks, the presence of posterior zone 1 disease, the occurrence of preretinal haemorrhages, history of sepsis, oxygen therapy, mechanical ventilation, respiratory distress syndrome, and patent ductus arteriosus [5, 6].

The cornerstone of surgical intervention in stage 4 ROP is a combination of anatomical retinal reattachment and recession of vascular activity to achieve the best functional outcome [7–9]. Several surgeries have been described as the treatment of choice for advanced-stage ROP, including scleral buckling and vitrectomy with or without the addition of endolaser. Previous studies have shown that the outcome success rate was highest in stage 4A ROP. Moreover, stage 4B ROP had a moderate success rate with sufficient visual outcomes.

Scleral buckling for stage 4A ROP is an effective method of preventing further progression of retinal detachment. Hinz et al. believed that the effect of the buckle not only relieves vitreoretinal traction and plays a role in preventing ischemia in the detached retina, thereby altering the natural progression of the disease [10]. Papageorgiou et al. additionally described improved visual outcomes in patients treated with scleral buckling [9]. In patients with temporal side retinal detachment only, segmental scleral buckling provides adequate results [11]. Segmental scleral buckling reportedly induces less myopia and does not require buckle removal and has the potential to significantly improve the treatment of stage 4A ROP [12].

Scleral buckling appears to reduce the progression from stage 4 to stage 5 ROP. While the anatomical success rate was excellent, but the visual results remained challenging in these cases [13].

As is well understood, scleral buckling, with or without external cryotherapy and subretinal fluid drainage, has been successful in achieving retinal reattachment in ROP patients [14]. However, the spontaneous resorption of subretinal fluid following this procedure has not been extensively studied. Studies on scleral buckling for other retinal have reported delayed absorption of subretinal fluid [14, 15] and compared the effectiveness of different drainage techniques [15]. Other studies have also shown that delayed absorption of subretinal fluid, which occurs after scleral buckling surgery, can be linked to factors such as marginally liquefied vitreous and delayed fluid reabsorption [16].

The absorption of subretinal fluid following scleral buckling surgery has been extensively analyzed using multifactorial approaches, underscoring its pivotal role in the management of rhegmatogenous retinal detachment [17]. These findings suggest that further research is needed to understand the factors influencing the resorption of subretinal fluid after scleral buckling in stage 5 ROP.

Delayed subretinal fluid resorption in stage 5 ROP is considered to be a multifaceted phenomenon that is intricately linked with elevated levels of vascular endothelial growth factor (VEGF) in the subretinal fluid. Lashkari et al. reported elevated VEGF levels in the subretinal fluid of eyes affected by active stage 4 ROP, contrasting with diminished levels in stage 5 [18]. This finding was further supported by Sonmez et al., who observed a significant increase in vitreous VEGF levels in eyes with vascularly active stage 4 ROP [19]. Sato et al. [20] also reported a significant increase in vitreous VEGF levels in eyes with stage 4 ROP, particularly in cases of moderate vascular activity. Similarly, Pieh et al. observed a substantial reduction in VEGF-A levels in stage 5 ROP cases [18]. This suggests that VEGF levels may play a potential role in the delayed resorption of subretinal fluid in stage 5 ROP. However, in our case, we assumed that anti-VEGF therapy could potentially worsen the traction, which is why we did not administer it.

Wang et al. demonstrated that targeted knockdown of VEGF-A in Müller cells reduced intravitreal neovascularization in a rat model of ROP. This suggests that decreased VEGF-A levels may be associated with reduced neovascularization in ROP. However, the relationship between VEGF-A levels and disease progression is complex. This highlights the need for further research to fully understand the bigger role of VEGF-A in ROP beside its therapeutic function [21].

## Conclusion

Patients with multiple comorbidities are more likely to have an aggressive form of ROP. Stage 5 ROP is a rare but devastating condition that is extremely difficult to treat, even under optimal conditions. Surgical procedures such as buckle surgery can achieve anatomical success in these selected cases, although functional visual acuity usually remains severely impaired. This case is remarkable because it shows that even in stage 5, slow but complete resorption of the subretinal fluid and thus reattachment of the retina is possible after buckle surgery. This highlights the importance of a wait-and-see approach, particularly when persistent subretinal fluid is present following buckle surgery, even in ROP patients. While the final visual function may remain low, anatomical retinal reattachment is valuable as it enables normal orbital and facial development. This rare case therefore provides valuable information for decision-making regarding surgical therapies for advanced stages of ROP.

## Data Availability

Data sharing is not applicable to this article as no datasets were generated or analysed during the current study.

## References

[1] Gupta K, et al. A quantitative severity scale for retinopathy of prematurity using deep learning to monitor disease regression after treatment. JAMA Ophthalmol. 2019;137(9):1029–36. 31268499 10.1001/jamaophthalmol.2019.2442 PMC6613298

[2] Sen P, Jain S, Bhende P. Stage 5 retinopathy of prematurity: an update. Taiwan J Ophthalmol. 2018;8(4):205–15. 30637192 10.4103/tjo.tjo_61_18 PMC6302569

[3] Karacorlu M, et al. Long-term functional results following vitrectomy for advanced retinopathy of prematurity. Br J Ophthalmol. 2017;101(6):730–4. 27635064 10.1136/bjophthalmol-2016-309198

[4] Rajan RP, et al. Clinico-demographic profile and outcomes of 25-gauge vitrectomy in advanced stage 5 retinopathy of prematurity. Graefes Arch Clin Exp Ophthalmol. 2021;259(7):1695–701. 33409680 10.1007/s00417-020-05063-2

[5] Barry GP, et al. Short-term retinal detachment risk after treatment of type 1 retinopathy of prematurity with laser photocoagulation versus intravitreal bevacizumab. J Aapos. 2019;23(5):e2601–4. 10.1016/j.jaapos.2019.05.013 31513902

[6] Bourla DH, et al. Association of systemic risk factors with the progression of laser-treated retinopathy of prematurity to retinal detachment. Retina. 2008;28(3 Suppl):S58–64. 18317347 10.1097/IAE.0b013e31815075b0

[7] Hansen ED, Hartnett ME. A review of treatment for retinopathy of prematurity. Expert Rev Ophthalmol. 2019;14(2):73–87. 31762784 10.1080/17469899.2019.1596026 PMC6874220

[8] Futamura Y, et al. Buckling surgery and supplemental intravitreal bevacizumab or photocoagulation on stage 4 retinopathy of prematurity eyes. Jpn J Ophthalmol. 2015;59(6):378–88. 26265249 10.1007/s10384-015-0401-5

[9] Papageorgiou E, et al. Scleral buckling surgery for stage 4A and 4B retinopathy of prematurity in critically ill neonates. Int Ophthalmol. 2022;42(4):1093–100. 34724137 10.1007/s10792-021-02095-3

[10] Hinz BJ, de Juan E Jr., Repka MX. Scleral buckling surgery for active stage 4A retinopathy of prematurity. Ophthalmology. 1998;105(10):1827–30. 9787350 10.1016/S0161-6420(98)91023-5

[11] Chuang YC, Yang CM. Scleral buckling for stage 4 retinopathy of prematurity. Ophthalmic Surg Lasers. 2000;31(5):374–9. 11011705

[12] Zhong Y, et al. Evaluation of segmental scleral buckling surgery for stage 4A retinopathy of prematurity in China. Front Med (Lausanne). 2022;9:969861. 35991664 10.3389/fmed.2022.969861 PMC9381871

[13] Ratanasukon M, et al. Outcomes of scleral buckling for stage 4 retinopathy of prematurity in Thai children. J Med Assoc Thai. 2006;89(10):1659–64. 17128841

[14] Greven C, Tasman W. Scleral buckling in stages 4B and 5 retinopathy of prematurity. Ophthalmology. 1990;97(6):817–20. 2374687 10.1016/s0161-6420(90)32506-x

[15] Malagola R, et al. Drainage of subretinal fluid during scleral buckling surgery for rhegmatogenous retinal detachment. G Chir. 2015;36(3):106–11. 26188754 PMC4511038

[16] Tee JJ, Veckeneer M, Laidlaw DA. Persistent subfoveolar fluid following retinal detachment surgery: an SD-OCT guided study on the incidence, aetiological associations, and natural history. Eye (Lond). 2016;30(3):481–7. 26742870 10.1038/eye.2015.270 PMC4791708

[17] Long K, et al. Multifactor analysis of delayed absorption of subretinal fluid after scleral buckling surgery. BMC Ophthalmol. 2021;21(1):86. 33588767 10.1186/s12886-021-01853-2 PMC7885473

[18] Lashkari K, et al. Vascular endothelial growth factor and hepatocyte growth factor levels are differentially elevated in patients with advanced retinopathy of prematurity. Am J Pathol. 2000;156(4):1337–44. 10751359 10.1016/S0002-9440(10)65004-3 PMC1876877

[19] Sonmez K, et al. Vitreous levels of stromal cell-derived factor 1 and vascular endothelial growth factor in patients with retinopathy of prematurity. Ophthalmology. 2008;115(6):1065–e10701. 18031819 10.1016/j.ophtha.2007.08.050

[20] Sato T, et al. Vitreous levels of erythropoietin and vascular endothelial growth factor in eyes with retinopathy of prematurity. Ophthalmology. 2009;116(9):1599–603. 19371954 10.1016/j.ophtha.2008.12.023

[21] Wang H, et al. Short hairpin RNA-mediated knockdown of VEGFA in Müller cells reduces intravitreal neovascularization in a rat model of retinopathy of prematurity. Am J Pathol. 2013;183(3):964–74. 23972394 10.1016/j.ajpath.2013.05.011 PMC3763762

